# Draft genomes of two contemporary strains of *Babesia divergens*

**DOI:** 10.1128/mra.00898-24

**Published:** 2024-12-13

**Authors:** Ternenge T. Apaa, Ben P. Jones, Adam M. Blanchard, Nicholas Johnson

**Affiliations:** 1 Vector Borne Diseases, Virology Department, Animal Plant and Health Agency, Addlestone, Surrey, United Kingdom; 2 Rabies and Viral Zoonoses, Virology Department, Animal Plant and Health Agency, Addlestone, Surrey, United Kingdom; 3 School of Veterinary Medicine and Science, University of Nottingham, Sutton Bonington, United Kingdom; 4 Faculty of Health and Medical Sciences, University of Surrey, Guildford, Surrey, United Kingdom; University of California Riverside, Riverside, California, USA

**Keywords:** *Babesia divergens*, genomes, direct DNA sequencing, Great Britain

## Abstract

*Babesia divergens*, *a* major cause of bovine babesiosis with zoonotic potential, was analyzed through genomes Bdiv23B and Bdiv24B following Illumina sequencing of DNA extracted from PCR-positive cattle blood. The genomes comprised 3888 and 4032 predicted coding sequences, respectively, comparable to the reference genome, Rouen 1987, highlighting genomic consistency across isolates.

## ANNOUNCEMENT

Babesia infection can lead to significant disease in both humans and domestic animals. Bovine babesiosis, commonly called redwater fever, causes febrile illness, anorexia, hemolytic anemia, dehydration, and diarrhea (1, 2). Disease results from high parasitic loads destroying erythrocytes, releasing hemoglobin into the urine that can be fatal if not treated promptly. Genomic analysis has emerged as a powerful tool for understanding pathogen biology, pathogenicity, and evolutionary relationships (3, 4). Previously, it was necessary to culture *Babesia divergens* prior to DNA preparation and sequencing, a process that is time-consuming. To avoid this limitation, we have derived *B. divergens* genomes directly from an infected cattle blood sample.

*B. divergens* was detected in two cattle blood samples by PCR (5). Prior to extraction, red blood cells were lysed with 0.15% saponin prepared in 1× phosphate-buffered saline (PBS) (150 mg: 99.85 mL 1× PBS) and the parasite preparation washed with PBS. Total DNA was extracted using the AllPrep DNA/RNA Kit (QIAgen, UK) following the manufacturer’s instruction. Fragmented DNA libraries were prepared for both samples using the Nextera XT Kit (Illumina, Cambridge, UK) and sequenced using NovaSeq (Illumina) to generate 150 base pair-end reads. Reads were quality-filtered and trimmed using fastp v0.23.1 (6), then classified taxonomically with Kraken2 v2.1.2 protozoa and fungal reference database (accessed 10 March 2023) (7) to confirm *Babesia* presence. First, depletion of host reads from all data sets was conducted by mapping reads to a cattle reference genome, ARS-UCD2.0 (accession no. GCF002263795.3) and extracting unmapped reads. Second, recovered reads not mapped to cattle genome were then mapped to concatenated *B. divergens* reference genome assemblies available in the National Center for Biotechnology Information (NCBI) database (1987:GCA001077455.2 and 1802A:GCA018398725). Third, reads mapped to concatenated *B. divergens* genomes were extracted for *de novo* genome assembly using SPAdes Genome Assembler v3.15.5 (8). Mapping and extraction of both unmapped and mapped reads data sets (steps 1–3) were conducted using bowtie2 v2.5.2 (9) and samtools v1.9 (10). Assembled contigs were also classified against the NCBI Blastn Database (accessed 19 December 2023) using SprayNPray v1.0 (11). Filtering and extraction of contigs ≥ 500 bp from the resulting assemblies were conducted using SeqKit v2.7.0 (12). Quality evaluation in comparison with *B. divergens* reference genomes Rouen 1987 was performed on assembled genomes using QUAST v5.2.0 (13) (Table 1). All bioinformatics analyses were conducted using default parameters. This approach allowed genome derivation directly from infected cattle blood DNA without requiring passage in purified bovine red blood cells, enhancing our understanding of the parasite’s genetic makeup.

**TABLE 1:** Genome comparison of *Babesia divergens* genomes generated by this study and the reference Rouen 1987^*a*^

Parameter	Bdiv23B	Bdiv24B	Rouen 1987^a^
Genome size (Mb)	8.15	8.30	9.7
GC% content	45.7	45.6	45.5
No. of contigs	530	725	459
Contig N50 (kb)/L50	146.42/17	148.82/19	123.5/22
Total reads sequenced (M)	59.8	64.8	NA
Total reads (M) used in genome assembly	28.82	28.96	NA
Reads mapped to reference (M)	28.61	28.61	NA
Avg. coverage depth	464	459	353
GenBank accession number	JBDXMZ000000000	JBDXMY000000000	GCA001077455.2

^*a*^ *B. divergens* (14); NA: not applicable; M: total sequences in millions.

## Data Availability

This Whole Genome Shotgun (WGS) project has been deposited at DDBJ/ENA/GenBank under the Bioproject PRJNA1091155, accession numbers JBDXMZ000000000 (version JBDXMZ000000000.1; SRA: SRR28427025) and JBDXMY000000000 (version JBDXMY000000000.1; SRA: SRR28427024). A set of bioinformatic scripts used in this analysis has been deposited in GitHub to ensure reproducibility and consistency (https://github.com/Ter-lab/Babesia-divergens-genome-analysis/tree/main).
